# Single-cell transcriptome *in silico* analysis reveals conserved regulatory programs in macrophages/monocytes of abdominal aortic aneurysm from multiple mouse models and human

**DOI:** 10.3389/fcvm.2022.1062106

**Published:** 2023-01-09

**Authors:** Shiyong Wu, Shibiao Liu, Baoheng Wang, Meng Li, Chao Cheng, Hairong Zhang, Ningheng Chen, Xueli Guo

**Affiliations:** ^1^Department of Vascular Surgery, The First Affiliated Hospital of Zhengzhou University, Zhengzhou, Henan, China; ^2^Center for Genome Analysis, Wuhan Ruixing Biotechnology Co., Ltd., Wuhan, China; ^3^Department of Colorectal and Anal Surgery, The First Affiliated Hospital of Zhengzhou University, Zhengzhou, Henan, China

**Keywords:** abdominal aortic aneurysm, single-cell transcriptome analysis, macrophages/monocytes, single-cell RNA sequencing, *in silico* analysis

## Abstract

Abdominal aortic aneurysm (AAA) is a life-threatening disease and there is currently a lack of effective treatment to prevent it rupturing. ScRNA-seq studies of AAA are still lacking. In the study, we analyzed the published AAA scRNA-seq datasets from the mouse elastase-induced model, CaCl_2_ treatment model, Ang II-induced model and human by using bioinformatic approaches and *in silico* analysis. A total of 26 cell clusters were obtained and 11 cell types were identified from multiple mouse AAA models. Also, the proportion of Mφ/Mo increased in the AAA group and Mφ/Mo was divided into seven subtypes. There were significant differences in transcriptional regulation patterns of Mφ/Mo in different AAA models. The enrichment pathways of upregulated or downregulated genes from Mφ/Mo in the three mouse datasets were different. The actived regulons of Mφ/Mo had strong specificity and the repressed regulons showed high consistency. The co-upregulated genes as well as actived regulons and co-downregulated genes as well as repressed regulons were closely correlated and formed regulatory networks. Mφ/Mo from human AAA dataset was divided into five subtypes. The proportion of three macrophage subpopulations increased but the proportion of two monocyte subpopulations decreased. In the AAA group, the upregulated or downregulated genes of Mφ/Mo were enriched in different pathways. After further analyzing the genes in Mφ/Mo of both mouse and human scRNA-seq datasets, two genes were upregulated in the four datasets, *IL-1B* and *THBS1*. In conclusion, *in silico* analysis of scRNA-seq revealed that Mφ/Mo and their regulatory related genes as well as interaction networks played an important role in the pathogenesis of AAA.

## Introduction

Abdominal aortic aneurysm (AAA), a cardiovascular disease with serious complications, is charactered by permanent local dilation of the abdominal aortic wall that exceeds 50% of the normal blood vessel diameter (1). As the disease worsens and the inner diameter of aorta dilates, the risk of AAA rupture increases (2). Over time, AAA can grow in size and rupture, causing life-threatening bleeding. Patients with AAA are usually asymptomatic until a catastrophic rupture occurs (3). Inflammatory cell infiltration, neovascularization, and the production as well as activation of various proteases as well as cytokines contributed to the development of AAA (4). Inflammatory processes played a critical role in AAA and significantly affected many determinants of aortic wall remodeling (5, 6). Various inflammatory cell types in AAA, such as macrophages, CD4^+^ T cells, and B cells, had great importance in the pathological aortic wall through phenotypic regulation (7). In addition, continuous crosstalk between various cells also affected the occurrence and development of AAA (8, 9). Therefore, the study of cell heterogeneity in the development of AAA may be a breakthrough to further understand its pathogenesis and develop targeted drugs. However, the relevant studies are still scarce.

The advances in the pathophysiology of AAA partly depend on the development and application of effective animal AAA models that replicate the key aspects of human. The basic premise of these animal models is that they share the same biochemical and cellular mechanisms as human possesses. Mouse AAA models have been widely used to study the occurrence and progression of AAA, including spontaneous aneurysm formation, drug-induced aneurysm, surgically induced aneurysm, genetic manipulation, chemical induction, and dietary models. Chemical methods included intracavity infusion of elastase, perivascular incubation of calcium chloride (CaCl_2_), and subcutaneous infusion of angiotensin II (Ang II) (10). Validation of mouse AAA models will provide insights into the mechanism of progression of human AAA. However, in mouse AAA models induced by different ways, what are the differences as well as similarities of gene expression patterns in different cell clusters and what molecular mechanisms are common to human AAA remains unclear.

The main pathological features of AAA included extracellular matrix remodeling that related to degeneration and loss of vascular smooth muscle cells (VSMCs), accumulation and activation of inflammatory cells (11). MMP9 derived from macrophages was a key factor in the degradation of extracellular matrix and crucial for the development of AAA (12). Different monocytes and macrophages subpopulations played a key and differential role in the initiation, progression, and healing of AAA process (5). The specific role of macrophages/monocytes (Mφ/Mo) accumulation in AAA remains unclear. The human aneurysm tissue showed numerous infiltrating macrophages (5). Multiple studies have shown that multiple genes mediated the development or suppression of AAA by regulating macrophages (13–15). For example, *IL-1*β and *TNF*-α influenced the formation of AAA through differential effects on macrophage polarization (13). Histone demethylase JMJD3 induced *NF*κ*B*-mediated inflammatory gene transcription in infiltrating aortic macrophages. Targeted inhibition of *JMJD3* significantly reduced AAA amplification and attenuated macrophage-mediated inflammation *in vivo* (14). Chemokine *CCL7* contributed to Ang II-induced AAA by promoting M1 phenotype of macrophages through *CCR1/JAK2/STAT1* signaling pathway (15). Macrophages can also work through crosstalk with other cells. For example, macrophage-derived netrin-1 promoted AAA formation by activating MMP3 in VSMCs (8). However, most of the existing research conclusion were based on a certain AAA model, and there was still a lack of comparison of the differences of macrophage transcriptional regulation modes in different mouse AAA models.

Previous sequencing technologies are to study the aggregation of cells, reflecting the average level of cell clusters, which cannot objectively reflect the information of the occurrence and development of diseases; but the information contained and expression level between cells vary greatly. Single-cell RNA sequencing (scRNA-seq) enabled the amplification and sequencing of whole transcriptome at the single-cell level. The principle was to amplify the trace amounts of whole transcriptome RNA from isolated single cells, then perform high-throughput sequencing, finally output the gene expression level of each cell through bioinformatics analysis. ScRNA-seq technology can reveal the overall level of gene expression status within a single cell, accurately reflect the heterogeneity among cells (16–18), reveal the diversity of immune cells in tissues (19), and build an interaction network among different cell populations (17). Therefore, single-cell transcriptome sequencing has been widely used to detect gene expression in different cell types during reproduction, development and disease occurrence, and to reveal the molecular mechanisms of the functions and effects of different cells in these processes.

Single-cell RNA sequencing provided a useful tool for studying the heterogeneity and dynamic regulation of AAA cells and much information on cell-specific gene expression profiles during the development and progression of AAA. Davis identified increased *JMJD3* in aortic Mφ/Mo by using scRNA-seq from human AAA tissue, leading to upregulation of inflammatory immune responses (14). Hadi found that macrophage derived netrin-1 promotes the formation of AAA by activating MMP3 in VSMCs through scRNA-seq of mouse AAA (8). Yang induced AAA in C57BL/6J mouse by perivascular application of CaCl_2_ for scRNA-seq, and analyzed the transcriptional profile and potential functional characteristics of populations in VSMCs, fibroblasts and macrophages (20). Yu demonstrated the key role of *Malat1* VSMCs in the occurrence and progression of AAA by scRNA-seq of Ang II-induced AAA treated with or without the inhibitor (21). Zhao demonstrated the heterogeneity and cellular response of VSMCs and Mφ/Mo in the progression of AAA by scRNA-seq of elastase-induced AAA (22). These scRNA-seq datasets provided insights into the pathogenesis of diseases and were rich resources for developing novel targeted therapy strategies. Based on these published datasets of different AAA mouse models and human AAA tissues, we hoped to uncover the conserved transcriptional regulatory patterns of macrophages during the development of AAA, and thereby elucidated the potential key roles of these genes in the pathogenesis of AAA.

## Materials and methods

### Retrieval and process of public data

Unique Molecular Identifier (UMI) count matrix for AAA and control sample scRNA-seq data of 4 datasets (14, 20–22) were downloaded from the GEO database. The UMI count matrix was converted from R package Seurat to Seurat objects (23) (version 4.0.4). Cells with UMI number < 500 or detected genes < 200 or those with mitochondrial-derived UMI counts of more than 10% were considered to be of low quality and were removed. Genes detected in less than 3 cells were removed for downstream analysis.

### ScRNA-seq data preprocessing and quality control

After quality control, the UMI count matrix was log normalized. Then top 2,000 variable genes were applied to create potential Anchors by using Seurat’s FindIntegrationAnchors function. Subsequently, IntegrateData function was applied to integrate data. In order to reduce the dimensionality of the scRNA-seq datasets, principal component analysis (PCA) was performed on the integrated data matrix. With Elbowplot function from Seurat, top 50 principal components (PCs) were applied to perform the downstream analysis. The main cell clusters were recognized with the FindClusters function of Seurat, with resolution set as default (res = 0.4). Finally, the cells aggregated into different cell types. Then they were visualized with t-distributed stochastic neighbor embedding (tSNE) or uniform manifold approximation and projection (UMAP) plots. For the gene markers of each cell clusters, we used the FindMarkers function in the Seurat package (version 4.0.4), and then we annotated cell types using previously published marker genes (24, 25).

### Differential gene expression analysis

The Seurat package FindMarkers/FindAllMarkers functions (one-tailed Wilcoxon rank sum test, *p*-values adjusted for multiple testing using the Bonferroni correction) were used to determine the differentially expressed genes (DEGs). When DEGs were calculated, the expression difference of all genes on the natural logarithmic scale was at least 0.5, and the adjusted *p*-value was less than 0.05.

### Transcription factor regulatory network analysis

The modules of transcription factor (TF) were recognized by the SCENIC (26) python workflow (version 0.11.2) using default parameters.^1^ A list of mouse TF genes was extracted from the resources of pySCENIC.^2^ Actived TFs were identified in the AUC matrix and differentially actived TFs were selected by using the FindAllMarkers function of the Seurat package. The networks of the modules with TFs and their target genes were visualized by Cytoscape (version 3.9.1).^3^

### Functional enrichment analysis

To sort out functional categories of genes, Gene Ontology (GO) terms and Kyoto Encyclopedia of Genes and Genomes (KEGG) pathways were identified using KOBAS 2.0 (27). Hypergeometric test and Benjamini-Hochberg FDR controlling procedure were applied to define the enrichment of each term.

### Other statistical analysis

The pheatmap package^4^ in R was used for performing the clustering based on Euclidean distance.

## Results

### ScRNA-seq analysis of abdominal aortic tissue from different mouse AAA models identified distinct cells types

**FIGURE 1:**
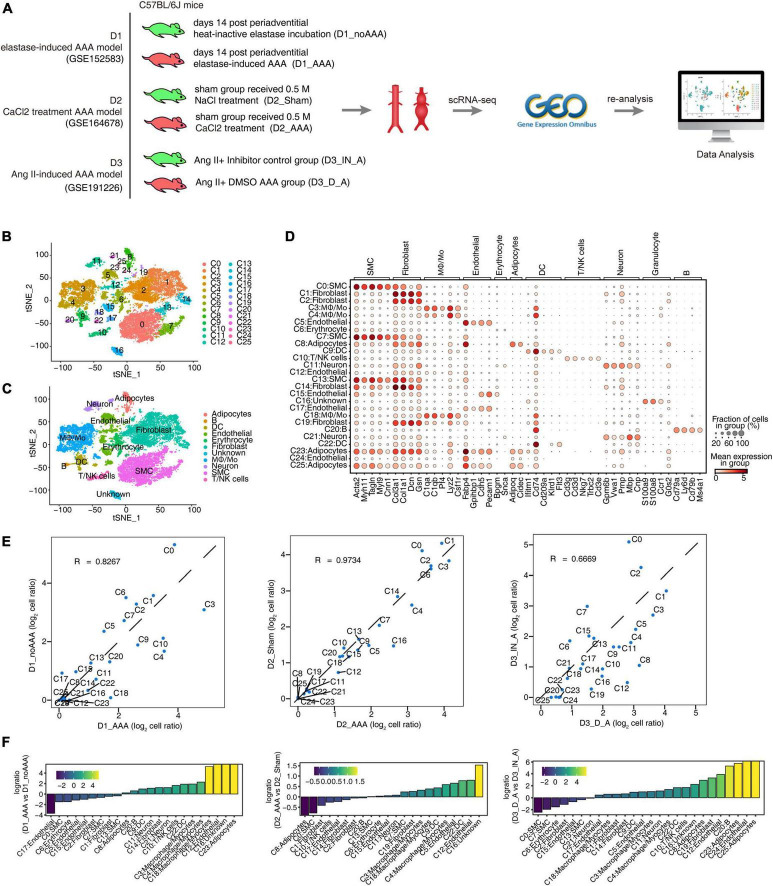
Single-cell RNA sequencing (ScRNA-seq) analysis of abdominal aortic tissue from different mouse abdominal aortic aneurysm (AAA) models identified distinct macrophages/monocytes (Mφ/Mo) types. **(A)** Schematic illustration of sample preparation and scRNA-seq data processing. **(B,C)** t-distributed stochastic neighbor embedding (tSNE) plot of composite single-cell transcriptomic profiles from all six abdominal aortic samples from three datasets. Colors indicated cell clusters along with annotations. Mφ/Mo, macrophage/monocyte cells; SMC, smooth muscle cells; DC, dendritic cells. **(D)** Dot plot showing expression of representative genes in each cell type. **(E)** Scatter plot comparing the proportions of cell populations of each cell type in two groups. **(F)** Rank order based on decreasing values of the relative frequency ratio between two sample groups.

In order to explore the differences and similarities of specifically key regulatory factors of aortic tissue cells in mouse AAA models constructed by different induction methods, we collected single-cell transcriptome datasets of the three published mouse AAA models: elastase induction (D1), CaCl_2_ induction (D2), and Ang II induction (D3). Each dataset included two samples of AAA group and control group (Figure 1A). Through data quality control procedure (Supplementary Table 1), the transcriptome map data of 22,391 single cells were obtained. The transcriptome expression matrix of them was normalized and analyzed by principal component dimension reduction. The top 50 PCs were selected for tSNE dimension reduction and visualization. After unbiased clustering analysis, 26 cell clusters were obtained (Figure 1B and Supplementary Figure 1A). Using the newly published SCTYPE software and combined with the reported cell markers in mouse artery tissue, 11 different cell types were identified (Figure 1C). As shown in Figure 1D and Supplementary Figure 1B, each cell type had specific representative genes and expression of top 3 marker genes. Most cell clusters were detected in three datasets, and only a few cell clusters, such as C24 and C25, were detected only in D3, because D3 had the largest number of cells (Supplementary Figure 1C). There was both consistency and difference in the variation trend of each cell subtype proportion in the three datasets (Figures 1E, F). The proportion of cell subtypes in AAA increased as follows: C3: Mφ/Mo, C4: Mφ/Mo, C9: DC, C11: Neuron, C12: Endothelial cell, C18: Mφ/Mo; the proportion of C0 (SMC) and C2 (Fibroblast) decreased in AAA; the proportion of other cell subtypes showed different trends in the three datasets (Figures 1E, F). The above results provided a panorama of cell compositions and changes in different mouse AAA models, and these cell subgroups also differed in functions (Supplementary Figure 1D).

### Single-cell analysis revealed complex Mφ/Mo heterogeneity and conserved regulated genes between AAA and control samples

**FIGURE 2:**
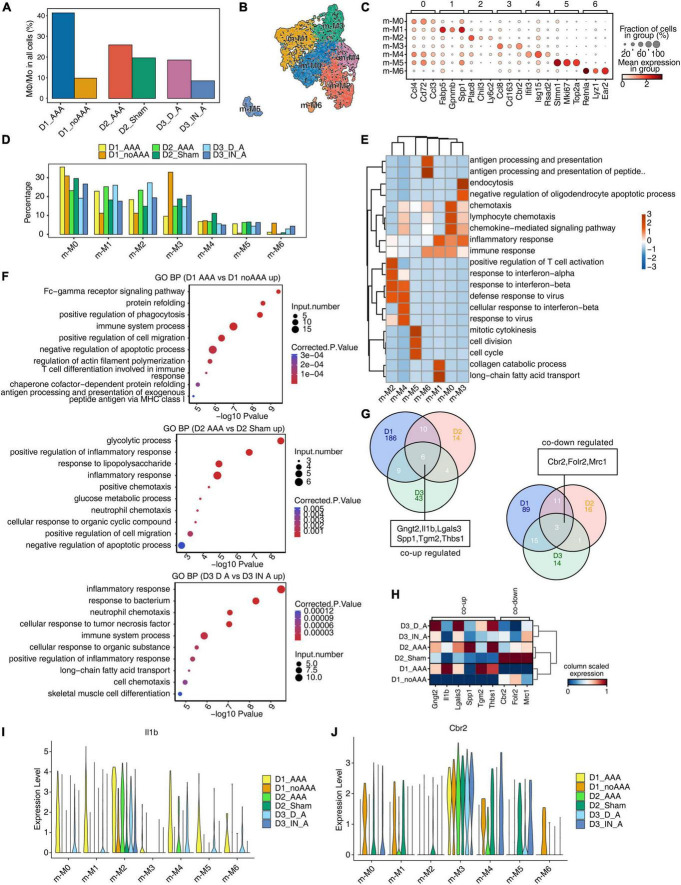
Single-cell analysis revealed complex macrophages/monocytes (Mφ/Mo) heterogeneity and conserved regulated genes between abdominal aortic aneurysm (AAA) and control samples. **(A)** Barplot represented the percentage of Mφ/Mo in total cells. **(B)** Uniform manifold approximation and projection (UMAP) plot of single-cell RNA sequencing (scRNA-seq) profile from Mφ/Mo separated into seven subtypes. Cells were colored according to different cell types. **(C)** Dot plot showing expression of top 3 markers in each subtype of Mφ/Mo. Color of dots represented the log fold change in each cluster comparing with other cells and dot size indicated percentage of cells in each cluster expressing the marker genes. **(D)** Bar plot comparing the proportions of cell populations of subtype of Mφ/Mo within each sample group. **(E)** Gene ontology enrichment analysis of biological processes of marker genes of each cell type. Top 3 terms were selected for each cluster and heatmap showed the enrichment *q*-value of these terms (scaled by column). **(F)** Gene ontology terms enriched in AAA versus in control Mφ/Mo for each dataset, respectively. The top 10 terms from upregulated genes are depicted as scatter plots displaying –log_10_ (*p*-value) and gene number. **(G)** Venn diagram showing the co-up (left) and co-down (right) regulated genes comparing AAA Mφ/Mo with control group from three datasets. **(H)** Unsupervised clustering heatmap showing relative expression (column scaled) levels of co-up genes and co-down genes in Mφ/Mo of each sample group. **(I,J)** Gene expression level of *Il-1b* **(I)** and *Cbr2* **(J)** were represented in the violin plot split by different sample groups.

Studies have shown that Mφ/Mo played a unique and important role in the occurrence and development of AAA in both patients and animal models (5, 14, 28). So, it is particularly important to further study the changes of Mφ/Mo in different mouse AAA models and the regulation of its related gene expression. On the whole, the proportion of Mφ/Mo increased in the AAA group compared with the control group, with a larger increase in D1 and D3 datasets and a smaller increase in D2 dataset (Figure 2A). It may be related to the shorter construction time of the AAA model in D2 dataset. We conducted secondary clustering of Mφ/Mo. A total of seven subtypes were generated (Figure 2B and Supplementary Figure 2A) and the top 3 marker genes of these subtypes were shown (Figure 2C). Taking Mφ/Mo as the overall background, it’s found that the changes of the relative proportions of Mφ/Mo subtypes in the three groups were highly dynamic and heterogeneous (Figure 2D and Supplementary Figure 2B). For example, the relative proportion of m-M0 type increased in AAA of D1 dataset and decreased in AAA of D2 and D3 datasets; the m-M1 as well as m-M2 types increased and m-M3 as well as m-M6 types decreased in AAA of the three datasets; the proportion of m-M4 and m-M5 varied in the three datasets (Figure 2D and Supplementary Figure 2B). The marker genes enriched functions of Mφ/Mo subtypes were also varied to different extent (Figure 2E). Further, we explored the changes in gene expressions in Mφ/Mo during the development of AAA. Compared with the control group, the enrichment functions of upregulated genes were different. But it was relatively consistent that inflammatory response, immune system and apoptotic process pathways were mainly enriched in the three datasets (Figure 2F). For downregulated genes, the enriched pathways were also significantly different in the three datasets (Supplementary Figure 2C). These results suggested that there were significant differences in the transcriptional regulation patterns of Mφ/Mo in different mouse AAA models. The intersection of upregulated and downregulated genes in the three datasets showed that six genes, *Gngt2*, *Il-1b*, *Lgals3*, *Spp1*, *Tgm2* as well as *Thbs1*, were upregulated and three genes, *Cbr2*, *Folr2* as well as *Mrc1*, were downregulated (Figure 2G). As shown in Figure 2H, the expression of these co-DEGs was showed in the three datasets, and their changes displayed certain heterogeneity in different Mφ/Mo subtypes (Figures 2I, J and Supplementary Figures 2D, E).

### Different AAA models had similar inhibition characteristics of transcription factors

**FIGURE 3:**
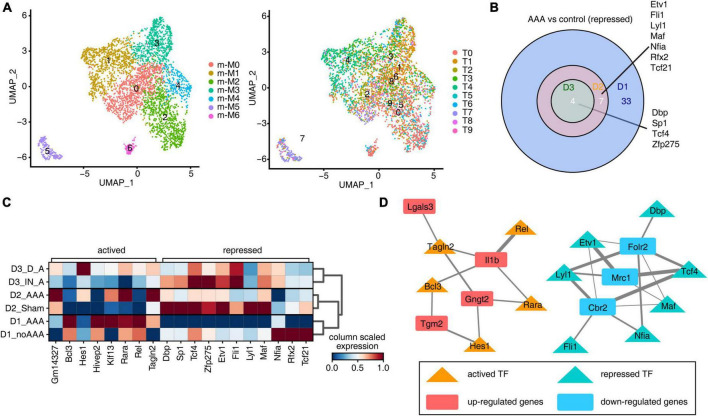
Different abdominal aortic aneurysm (AAA) models had similar inhibition characteristics of transcription factors. **(A)** Uniform manifold approximation and projection (UMAP) displayed the distributions of 10 cell types based on regulons (right panel). Colors indicated cell types. **(B)** Venn diagram showing the co-repressed regulons comparing AAA macrophages/monocytes (Mφ/Mo) with control group from three datasets. **(C)** Unsupervised clustering heatmap displayed the active states of co-actived and co-repressed regulons in each sample groups. Red colors indicated that the network was more activated, blue colors indicated that the network was more silenced. **(D)** Cytoscape showed the regulatory networks comprising transcription factors from panel **(C)** and their target co-differentially expressed genes (DEGs) underlying Mφ/Mo. Edges connected transcription factor-target gene pairs while nodes represented genes.

To explore the regulons of Mφ/Mo, we used pySCENIC to calculate regulon activity scores (RASs) in all Mφ/Mo and Seurat to construct the Mφ/Mo transcriptional regulation map. It’s observed that the clusters displayed by regulons were generally consistent with Mφ/Mo subtypes but showed certain specificity (Figure 3A). It was also observed that different subgroups showed highly specific and different activation of regulons (Supplementary Figure 3A). By further comparing the actived and repressed regulons in AAA and control groups, it’s found that the actived regulons had strong specificity, and only one co-actived regulon *Gm14327* was found in the AAA group of the three datasets (Supplementary Figure 3B). However, the repressed regulons were showed high consistency and a total of four regulons were detected in three datasets, including *Dbp*, *Sp1*, *Tcf4*, *Zfp275* (Figure 3B). As shown in Figure 3C, the co-actived and co-repressed regulons in the intersection of two or three databases showed different expression levels in the control group and the AAA group. In order to further confirm the regulatory functions of these regulons, we constructed regulatory networks of the co-varied regulons and co-DEGs. The co-upregulated genes as well as actived regulons and co-downregulated genes as well as repressed regulons were closely correlated (Figure 3D). These results suggested that co-varied regulons of Mφ/Mo had existed in different mouse AAA models and formed regulatory networks with co-DEGs, which played an important role in the occurrence and development of AAA.

### ScRNA-seq of human AAA showed the increase of macrophages but not monocytes

**FIGURE 4:**
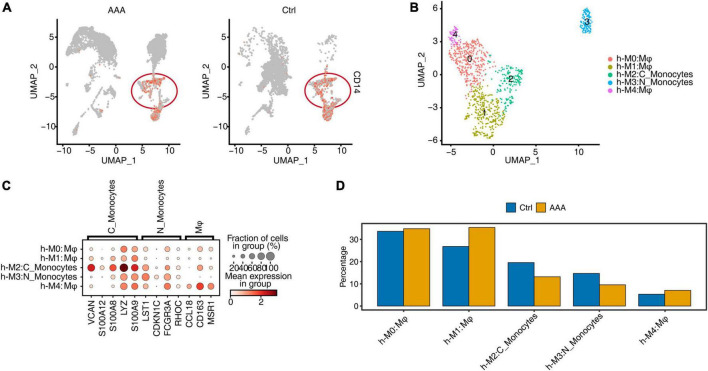
Single-cell RNA sequencing (ScRNA-seq) of human abdominal aortic aneurysm (AAA) showed the increase of macrophage cells but not monocytes. **(A)** Gene expression level of CD14 were represented in the uniform manifold approximation and projection (UMAP) plot split by human AAA and control group. **(B)** UMAP plot of scRNA-seq profile from macrophages/monocytes (Mφ/Mo) was separated into five subtypes. Cells were colored according to different cell types. C_Monocytes, classical monocytes; N_Monocytes, non-classical monocytes. **(C)** Dot plot showing expression of representative markers in each subtype of Mφ/Mo. Color of dots represented the log fold change in each cluster comparing with other cells and dot size indicated percentage of cells in each cluster expressing the marker genes. **(D)** Bar plot comparing the proportions of cell populations of subtype of Mφ/Mo within each sample group.

It may be difficult to obtain human AAA tissue samples. So far, few article has reported AAA single-cell data in humans. Model comparisons between animals and humans were critical to understand the pathogenicity and cell-specific regulatory factors they shared. We first downloaded the datasets from GEO and reanalyzed it (14). The clustering and annotation results showed that the cell composition of the AAA group and the control group had a very large specificity and divided into 23 cell clusters (Supplementary Figure 4A). Each cell type had specific representative genes and expression of top 3 marker genes (Supplementary Figure 4B). The relative proportions of cell clusters in human AAA and control group, and the proportions of cell populations within each sample group had obvious differences (Supplementary Figures 4C, D). Mφ/Mo, specifically expressing CD14, were extracted for clustering and annotation again (Figure 4A). Further, h-M0, h-M1, and h-M4 were annotated as macrophages, while h-M2 and h-M3 were annotated as monocytes (Figure 4B). Each subtype of Mφ/Mo had representative marker genes (Figure 4C). By comparing the relative proportion of these five subpopulations of Mφ/Mo, it’s found that the proportion of three macrophage subpopulations increased in AAA, while the proportion of two monocyte subpopulations decreased (Figure 4D). Also, different subpopulations of Mφ/Mo had their own specific GO enrichment of biological processes, which represented a different role in the development of AAA. The h-M0 was mainly enriched in synapse pruning and macrophage migration. The h-M1 was mainly enriched in immune response, inflammatory response and chemokine-mediated signaling pathway. The h-M3 was mainly enriched in immune response and neutrophil degranulation. The h-M2 and h-M4 were mainly enriched in neutrophil degranulation, but h-M4 was also enriched in endocytosis, regulation of macrophage migration (Supplementary Figure 4E).

### *IL-1B* and *THBS1* were most conserved regulated genes in Mφ/Mo during AAA development

**FIGURE 5:**
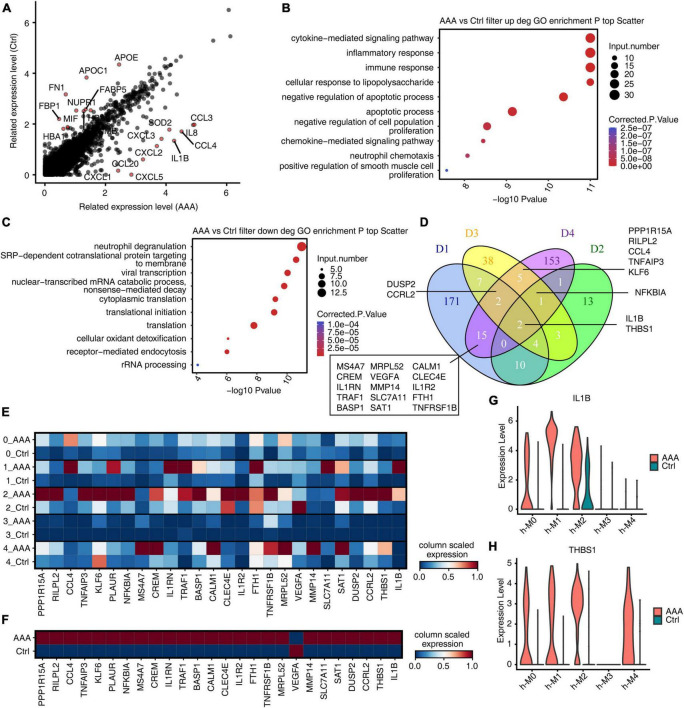
*IL-1B* and *THBS1* were most conserved regulated genes in macrophages/monocytes (Mφ/Mo) during abdominal aortic aneurysm (AAA) development. **(A)** Scatter plot displayed the differentially expressed genes in Mφ/Mo. Red colors indicated that the top 10 upregulated or downregulated genes. **(B)** Gene ontology terms of upregulated genes in AAA versus in control Mφ/Mo from human AAA dataset. The top 10 terms from upregulated genes were depicted as scatter plots displaying –log_10_ (*p*-value) and gene number. **(C)** Gene ontology terms of downregulated genes in AAA versus in control Mφ/Mo from human AAA dataset. The top 10 terms from upregulated genes were depicted as scatter plots displaying –log_10_ (*p*-value) and gene number. **(D)** Venn diagram showing the co-upregulated genes comparing AAA Mφ/Mo with control group from four datasets (D1–D3: mouse; D4: human). **(E)** Unsupervised clustering heatmap showing relative expression (column scaled) levels of upregulated genes at least in two datasets (include D4) split by human AAA and control group. **(F)** Same as panel **(E)** except that expression was showed in different subgroups of human AAA and control group. **(G, H)** Gene expression level of *IL-1B* and *THBS1* were represented in the violin plot split by human AAA and control group.

To compare with the mouse model, we firstly analyzed the genes of human AAA in Mφ/Mo that were differentially expressed from the control group (Figure 5A). The upregulated genes in the AAA group were significantly enriched in immune, inflammation, proliferation, and apoptosis related pathways (Figure 5B). The downregulated genes were mainly concentrated in neutrophil degranulation and translation related pathways (Figure 5C). The upregulated and downregulated genes in human AAA dataset and three mouse AAA datasets were intersected. Two genes, *IL-1B* and *THBS1*, were co-upregulated in the four datasets (Figure 5D). However, there were no co-downregulated genes, and only two downregulated genes (*APOE*, *FOS*) were found in D1 and human dataset (Supplementary Figure 5A). The genes that were upregulated in any two of the four datasets were extracted and displayed their expression in the human AAA and control samples. It’s found that most genes showed high expression in AAA group, indicating that the expression trends of these genes were consistent in mouse AAA model and human AAA tissues (Figures 5E, F). Similarly, we extracted downregulated genes detected in any two of the four datasets and displayed their expression in human AAA and the control group. Different from the upregulated genes, most downregulated genes showed a trend of high expression in human AAA tissues (Supplementary Figures 5B, C). As shown in Figures 5G, H, it’s found that *IL-1B* in macrophages subtypes h-M0 as well as h-M1, and *THBS1* in h-M0, h-M1 as well as h-M4 were significantly increased in AAA. These results suggested that *IL-1B* and *THBS1* were most conserved regulated genes and played a role in the involvement of Mφ/Mo in promoting the development of AAA both in mouse and humans.

## Discussion

Abdominal aortic aneurysm is a life-threatening disease and there is currently a lack of effective treatment to prevent it rupturing. The mammalian abdominal aorta is composed of a large number of multifunctional cell populations, and each cell cluster has a distinct relationship with AAA. Several studies have used the scRNA-seq technique to characterize the heterogeneity of vascular cells, including VSMCs (29), endothelial cells (ECs) (30), macrophages (31), and aortic advection cells (32) in healthy and atherosclerotic arteries. However, studies on the cellular heterogeneity and aneurysm-related transcriptional signatures during AAA development are still deficient. Here, based on the published scRNA-seq datasets of GSE152583, GSE164678, GSE191226, and GSE166676 from different mouse AAA models and human AAA tissues, we explored the cellular heterogeneity and conserved transcriptional regulation patterns of Mφ/Mo during AAA to elucidate potential critical roles of certain genes in the pathogenesis of AAA. In the study, 26 cell clusters were obtained and 11 different cell types were identified by their markers in the three AAA mouse models. The proportion variation and function of each cell subtype were both consistent and different (Figure 1 and Supplementary Figure 1). We further studied the heterogeneity of Mφ/Mo and conserved regulated genes between AAA and control samples. The proportion of Mφ/Mo increased in the AAA group. Mφ/Mo was divided into seven subtypes and the relative proportion changes of these subtypes were highly dynamic and heterogeneous. Moreover, the enrichment function of Mφ/Mo subtypes and DEGs had similarities as well as differences. In addition, there were co-downregulated genes, *Gngt2*, *Il-1b*, *Lgals3*, *Spp1*, *Tgm2* as well as *Thbs1*, and co-upregulated genes, *Cbr2*, *Folr2* as well as *Mrc1*, in the three datasets (Figure 2 and Supplementary Figure 2). Next, we explored the regulons of Mφ/Mo, the cluster groups displayed by regulons were generally consistent with Mφ/Mo subtypes but showed certain specificity. The activated regulons had strong specificity but the repressed regulons had shown high consistency. The co-upregulated genes as well as actived regulons and co-downregulated genes as well as repressed regulons were significantly correlated (Figure 3 and Supplementary Figure 3). The Mφ/Mo in human AAA tissue was divided into five subtypes. The proportion of three macrophage subpopulations increased in AAA, while the proportion of two monocyte subpopulations decreased. The different subpopulations had their own specific functions, indicating that scRNA-seq of human AAA showed the increase of macrophages but not monocytes (Figure 4 and Supplementary Figure 4). Finally, the upregulated and downregulated genes in the human AAA dataset and the three mouse AAA datasets were intersected. Two genes were co-upregulated in the four datasets, including *IL-1B* and *THBS1* (Figure 5 and Supplementary Figure 5). In summary, *in silico* analysis of scRNA-seq revealed that Mφ/Mo and its regulatory related genes as well as interaction networks played an important role in the pathogenesis of AAA.

Single-cell RNA sequencing is a contemporary and powerful technique for determining transcriptome gene profiles at the cellular level. ScRNA-seq has been recently used by many researchers to study the transcriptome profiles of aortic aneurysm tissues in humans and experimental animals at single-cell resolution (14, 20–22, 33). A comprehensive and unbiased genetic analysis by scRNA-seq can lead to a better understanding of cell-specific molecular signatures of AAA under physiological and pathophysiological conditions (34). At present, no single animal model can accurately reflect the full spectrum of human AAA pathophysiology (20). Therefore, further exploring the scRNA-seq datasets of different AAA models and human specimens will help better understand the pathogenesis of this disease from multiple perspectives and provide reference for clinical intervention. The scRNA-seq datasets (D1, D2, D3) from the three mouse AAA models have consistently demonstrated the heterogeneity of AAA tissue cells. Mφ/Mo infiltration in the adventitia of aneurysm tissue was a significant change during AAA progression. Mφ/Mo in the vessel wall had multiple functions, including amplification of the local inflammatory responses through secretion of proinflammatory cytokines, chemokines, and production of proteases and reactive oxygen species (5). Based on the scRNA-seq data herein, 26 cell clusters were obtained and 11 different cell types were identified. The proportion of different cell groups between AAA group and control group showed different trends. The proportion of cells such as Mφ/Mo, DC and neuron increased and the proportion of VSMC and fibroblast decreased in AAA. Monocytes and macrophages played a critical role in vascular injury and AAA formation. Macrophages were mainly derived from circulating monocytes and the main inflammatory cell types in AAA lesions (5). Monocytes adhesion, migration, and MMP-9 production all increased in AAA patients, leading to aneurysm expansion (35). Mφ/Mo were extracted for analysis and a total of 7 subtypes were defined, which expressed diverse factors and were highly dynamic and heterogeneous. For example, m-M0 highly expressed chemokines, such as *Ccl4*, *Cd72*, and *Ccl3*, which was related to chemotactic biological function. m-M3 highly expressed *Ccl8*, *Cd163*, and *Folr2*, which was related to endocytosis, oligodendrocyte apoptotic process, and inflammatory. The subtypes of Mφ/Mo and its expressed varied genes performed different biological functions and participated in mouse AAA. Further analysis revealed that some genes, *Gngt2*, *Il-1b*, *Lgals3*, *Spp1*, *Tgm2* as well as *Thbs1*, were upregulated in three datasets, which was consistent with numerous reports in the literature. *Il-1b* was a proinflammatory cytokine, but it effected AAA formation as well as macrophage polarization (13) and treatment with anti- *Il-1a* or anti-*Il-1b* mAb blocked LCWE-induced AAA formation (36). Transglutaminase 2 (*Tgm2*) expression and activity in AAA formation were enhanced and had a potential role of ECM protector in aortic walls during AAA remodeling (37). *THBSs* overexpression may affect the formation of matrix cells and inhibit the activity of matrix proteins, thus destroying the structure of extracellular matrix and affecting the AAA occurrence (38). Yang predicted commonly altered signaling pathways by using intercellular communication networks in different experimental AAA models and human AAA, with a particular focus on *THBS* signaling among different cell populations (39). These results suggested that these genes were vital in the occurrence and development of AAA and worth further exploring.

The co-upregulated genes as well as actived regulons and co-downregulated genes as well as repressed regulons from Mφ/Mo were significantly correlated and formed regulatory networks. There were differences in the number and regulatory correlation of co-varied regulons and co-DEGs interactions, which played important and different roles in the formation of AAA by affecting the function of Mφ/Mo. To explore variation of macrophages and monocytes in human AAA tissue samples, it’s found that the cell composition had a very large specificity between the AAA group and the control group, and immune cells were greatly amplified in the AAA group. The proportion of three macrophage subtypes (h-M0, h-M1, and h-M4) increased in AAA, while the proportion of two monocyte subtypes (h-M2 and h-M3) decreased. The pathways enriched in each subtype were also not same. The h-M0, h-M1, and h-M4 were mainly related to macrophage migration, immune inflammatory response and endocytosis; the h-M2 and h-M3 were mainly related to immune response and neutrophil degranulation, indicating that macrophages played a more important role than monocytes in human AAA. To further analyze the scRNA-seq datasets of Mφ/Mo from both mouse and human, it’s found that two genes, *IL-1B* and *THBS1*, were co-upregulated and no gene was co-downregulated in the four datasets. This fully demonstrated the critical role of *IL-1B* and *THBS1* in AAA, which is consistent with the previous discussion and related literature (36, 39). The formation mechanisms of AAA induced by elastase, CaCl_2_ as well as Ang II were different, and none of them can completely replace human AAA process. However, there were two upregulated DEGs, *IL-1B* and *THBS1*, in all three mouse AAA models and human AAA samples, indicating that they played a vital role in the commonly critical key link of AAA formation, which needed further exploration.

However, there were some limitations to our study. First of all, due to the reanalysis of the original datasets, the quality of them was also evaluated, but it was difficult to closely evaluate the reliability of the original samples, such as modeling quality, specimen collection process, and data sequencing. In the D3 dataset, no healthy control group data was provided, and the analysis of IN_A as a remission/rescue group instead of control group may have an effect on gene variation. Second, human AAA specimens were relatively few and existed individual differences, which required more human data support. Third, although we used the latest algorithms and other tools for evaluation, it may still cause some errors in the actual situation. Therefore, further studies were needed to provide more direct evidence for the role of Mφ/Mo in AAA.

In conclusion, this was the first study to compare the regulation of gene expression of Mφ/Mo in different mouse AAA models at the single-cell level. Our analysis revealed that co-DEGs of Mφ/Mo in the three mouse models played a critical role in the development of AAA. Moreover, we were the first to analyze and compare the transcriptional regulatory networks in different mouse AAA models. The co-varied regulons constituted the closely interaction regulatory networks with co-DEGs, regulating macrophage endocytosis, proliferation, and apoptosis. In addition, we determined the similarities and differences of the genes in the four datasets by comparing scRNA-seq datasets of three mouse AAA models and human AAA sample. In particular, *IL-1B* and *THBS1* were co-upregulated genes obtained from all four datasets and worthy of attention. These comparisons allowed us to show the cell classification, gene expression, and transcriptional regulatory networks in the current AAA models, which made us better grasp the similarities and differences of the models at the molecular level, and also provided a new idea for the development of animal models in line with human AAA as well as targeted interventions for AAA.

## Data availability statement

The original contributions presented in this study are included in the article/Supplementary material, further inquiries can be directed to the corresponding authors.

## Author contributions

SW, NC, and XG proposed and designed this research. SW wrote this manuscript. SW, HZ, CC, and SL participated in data analysis. CC, BW, and ML participated in the design of the study. SW, HZ, and XG reviewed and edited the manuscript. All authors contributed to the article and approved the submitted version.

## Footnotes

1
http://scenic.aertslab.org

2
https://github.com/aertslab/pySCENIC/tree/master/resources

3
https://cytoscape.org

4
https://cran.r-project.org/web/packages/pheatmap/index.html

## Supplementary material

Supplementary Figure 1 Single-cell RNA sequencing (ScRNA-seq) analysis of abdominal aortic tissue from different mouse abdominal aortic aneurysm (AAA) models identified distinct macrophages/monocytes (Mφ/Mo) types. **(A)** Uniform manifold approximation and projection (UMAP) plot split by different sample groups. **(B)** Dot plot showing expression of top 3 marker genes in each cell type. **(C)** Bar plot comparing the proportions of cell populations of each cell type within each sample group. **(D)** Gene ontology enrichment analysis of biological processes of top 100 marker genes of each cell type. Top 3 terms were selected for each cluster and heatmap shows the enrichment *q*-value of these terms (scaled by column).

Supplementary Figure 2 Single-cell analysis revealed complex macrophages/monocytes (Mφ/Mo) heterogeneity and conserved regulated genes between abdominal aortic aneurysm (AAA) and control samples. **(A)** Uniform manifold approximation and projection (UMAP) visualization of the Mφ/Mo split by different sample groups. **(B)** Rank order based on decreasing values of the relative frequency ratio between AAA and control sample group in three datasets. **(C)** Gene ontology terms of downregulated genes in AAA versus in control Mφ/Mo for each dataset, respectively. The top 10 terms from upregulated genes were depicted as scatter plots displaying −log_10_ (*p*-value) and gene number. **(D,E)** Gene expression level of *Spp1* **(D)** and *Mrc1* **(E)** were represented in the violin plot split by different sample groups.

Supplementary Figure 3 Different abdominal aortic aneurysm (AAA) models had similar inhibition characteristics of transcription factors. **(A)** Unsupervised clustering heatmap displayed the active states of cluster-specific regulons in each subtype of macrophages/monocytes (Mφ/Mo). Red colors indicated that the network was more activated, blue colors indicated that the network was more silenced. **(B)** Venn diagram showing the co-activated regulons comparing AAA Mφ/Mo with control group from three datasets.

Supplementary Figure 4 Single-cell RNA sequencing (ScRNA-seq) of human abdominal aortic aneurysm (AAA) showed the increase of macrophage cells but not monocytes. **(A)** Uniform manifold approximation and projection (UMAP) plot of composite single-cell transcriptomic profiles from all human AAA and control groups. Colors indicated cell clusters along with annotations. **(B)** Dot plot showing expression of top 3 marker genes in each cell type. **(C)** Stacked bar plot showing the relative proportions of cell clusters in human AAA and control group. **(D)** Bar plot comparing the proportions of cell populations of cell clusters within each sample group. **(E)** Gene ontology enrichment analysis of biological processes of marker genes of each subgroup of macrophage/monocyte cells. Top 3 terms were selected for each cluster and heatmap shows the enrichment *q*-value of these terms (scaled by column).

Supplementary Figure 5 *IL-1B* and *THBS1* were most conserved regulated genes in macrophages/monocytes (Mφ/Mo) during abdominal aortic aneurysm (AAA) development. **(A)** Venn diagram showing the co-downregulated genes comparing AAA Mφ/Mo with control group from four datasets (D1–D3: mouse; D4: human). **(B)** Unsupervised clustering heatmap showing relative expression (column scaled) levels of downregulated genes at least in two datasets split by human AAA and control group. **(C)** Unsupervised clustering heatmap showing relative expression (column scaled) levels of downregulated genes at least in two datasets showed in panel **(A)**, cells were split by different subgroups of human AAA and control group.

Supplementary Table 1 Quality control form of datasets.
