# Supplementary figures and images for: Single-cell transcriptome in silico analysis reveals conserved regulatory programs in macrophages/monocytes of abdominal aortic aneurysm from multiple mouse models and human
Source: Front Cardiovasc Med. 2023 Jan 9;9:1062106. doi: 10.3389/fcvm.2022.1062106 (PMC9868255; doi:10.3389/fcvm.2022.1062106)

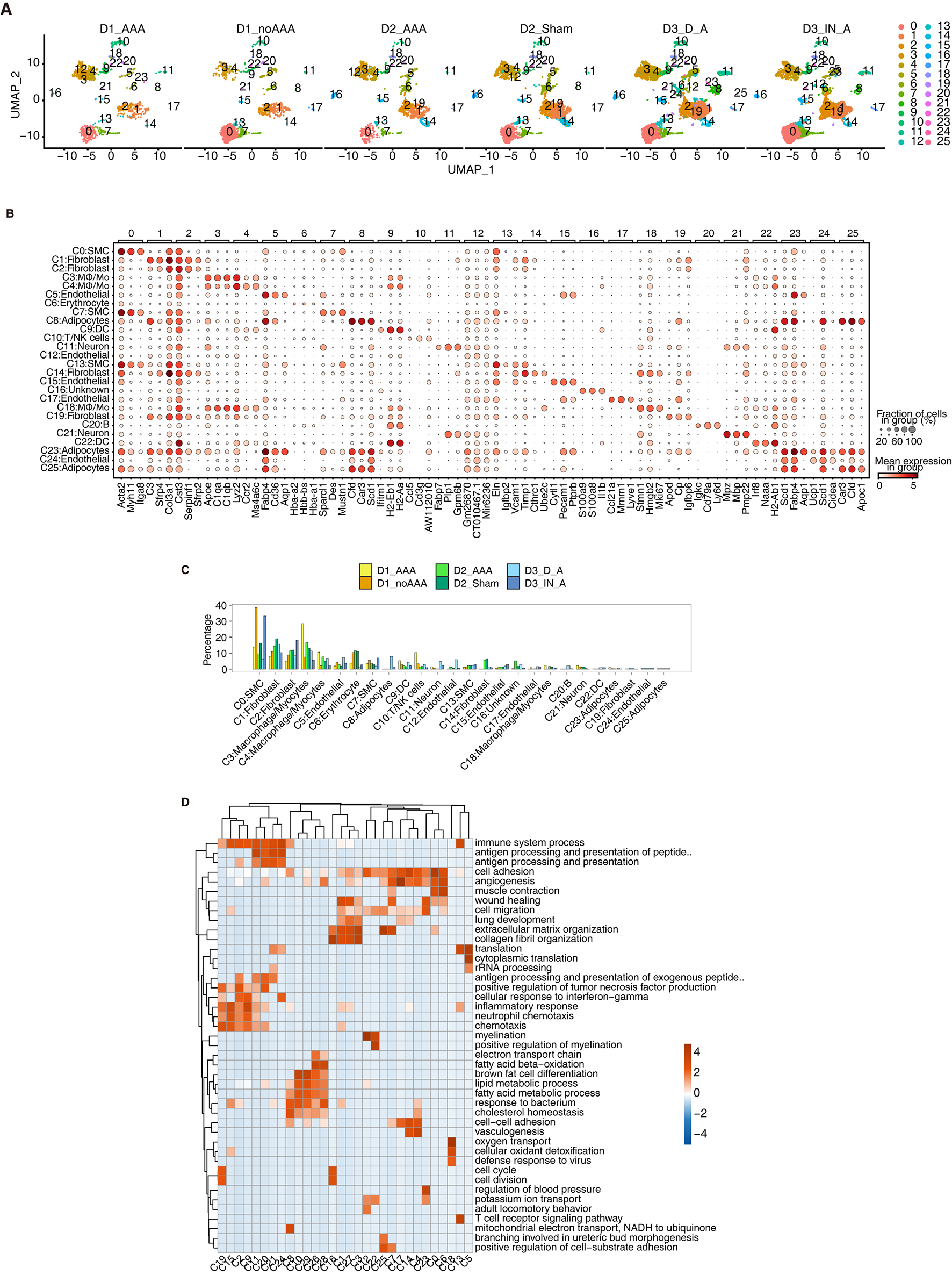

Supplement: Supplementary Figure 1 — Single-cell RNA sequencing (ScRNA-seq) analysis of abdominal aortic tissue from different mouse abdominal aortic aneurysm (AAA) models identified distinct macrophages/monocytes (Mφ/Mo) types. (A) Uniform manifold approximation and projection (UMAP) plot split by different sample groups. (B) Dot plot showing expression of top 3 marker genes in each cell type. (C) Bar plot comparing the proportions of cell populations of each cell type within each sample group. (D) Gene ontology enrichment analysis of biological processes of top 100 marker genes of each cell type. Top 3 terms were selected for each cluster and heatmap shows the enrichment q-value of these terms (scaled by column). [file Image_1.TIF]

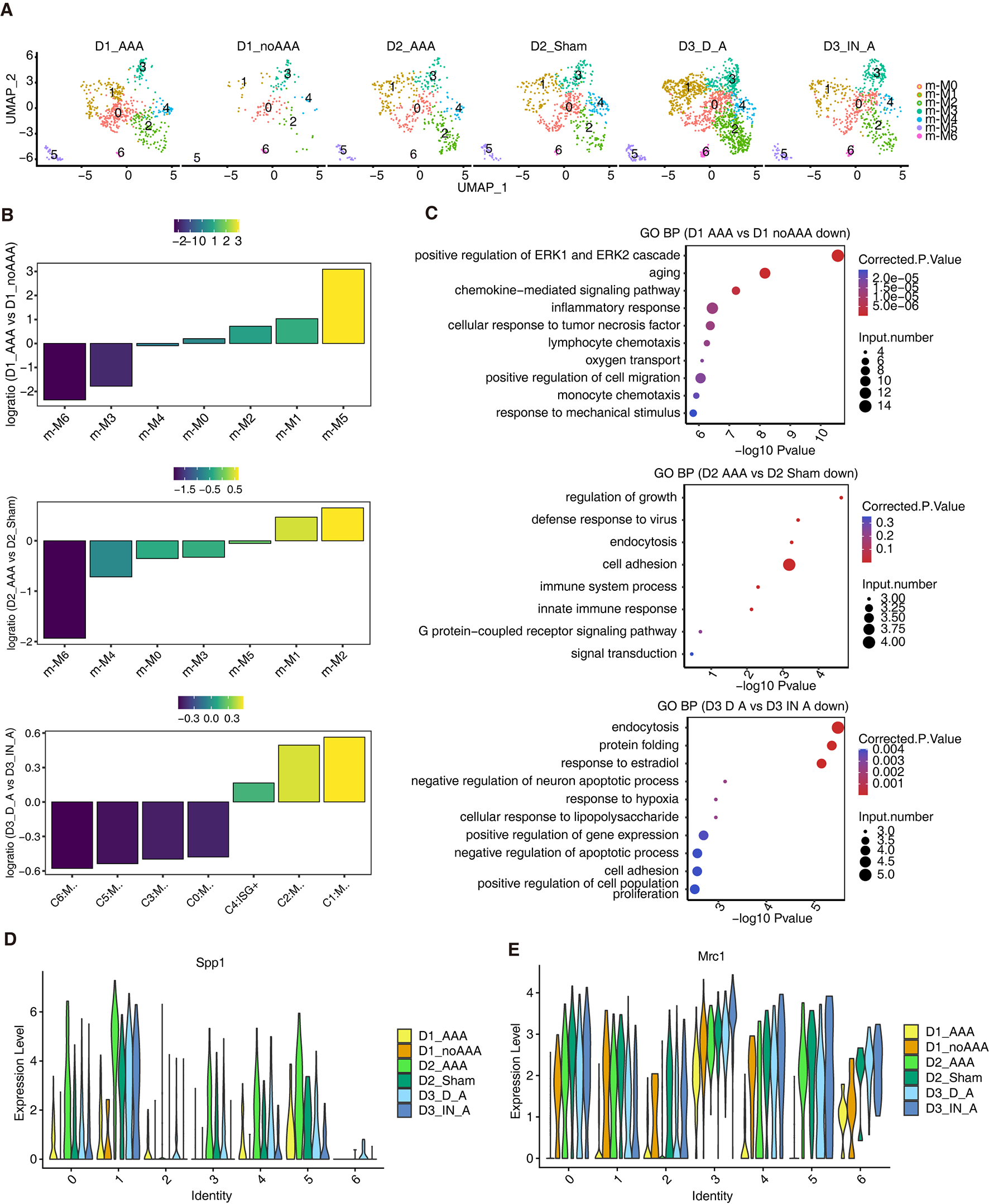

Supplement: Supplementary Figure 2 — Single-cell analysis revealed complex macrophages/monocytes (Mφ/Mo) heterogeneity and conserved regulated genes between abdominal aortic aneurysm (AAA) and control samples. (A) Uniform manifold approximation and projection (UMAP) visualization of the Mφ/Mo split by different sample groups. (B) Rank order based on decreasing values of the relative frequency ratio between AAA and control sample group in three datasets. (C) Gene ontology terms of downregulated genes in AAA versus in control Mφ/Mo for each dataset, respectively. The top 10 terms from upregulated genes were depicted as scatter plots displaying −log10 (p-value) and gene number. (D,E) Gene expression level of Spp1 (D) and Mrc1 (E) were represented in the violin plot split by different sample groups. [file Image_2.TIF]

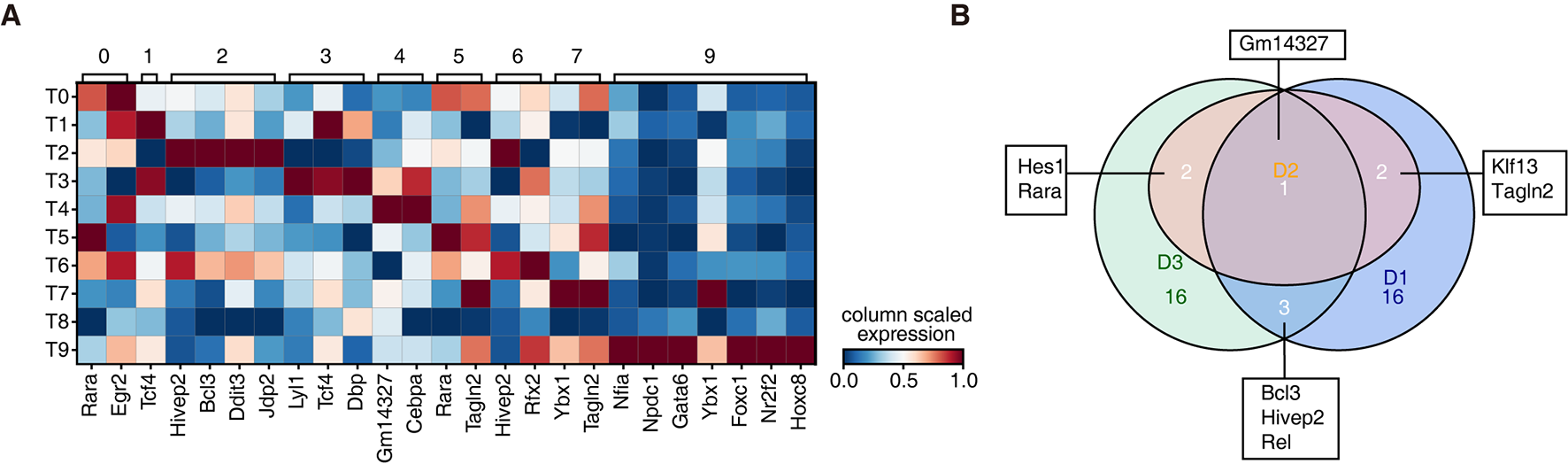

Supplement: Supplementary Figure 3 — Different abdominal aortic aneurysm (AAA) models had similar inhibition characteristics of transcription factors. (A) Unsupervised clustering heatmap displayed the active states of cluster-specific regulons in each subtype of macrophages/monocytes (Mφ/Mo). Red colors indicated that the network was more activated, blue colors indicated that the network was more silenced. (B) Venn diagram showing the co-activated regulons comparing AAA Mφ/Mo with control group from three datasets. [file Image_3.TIF]

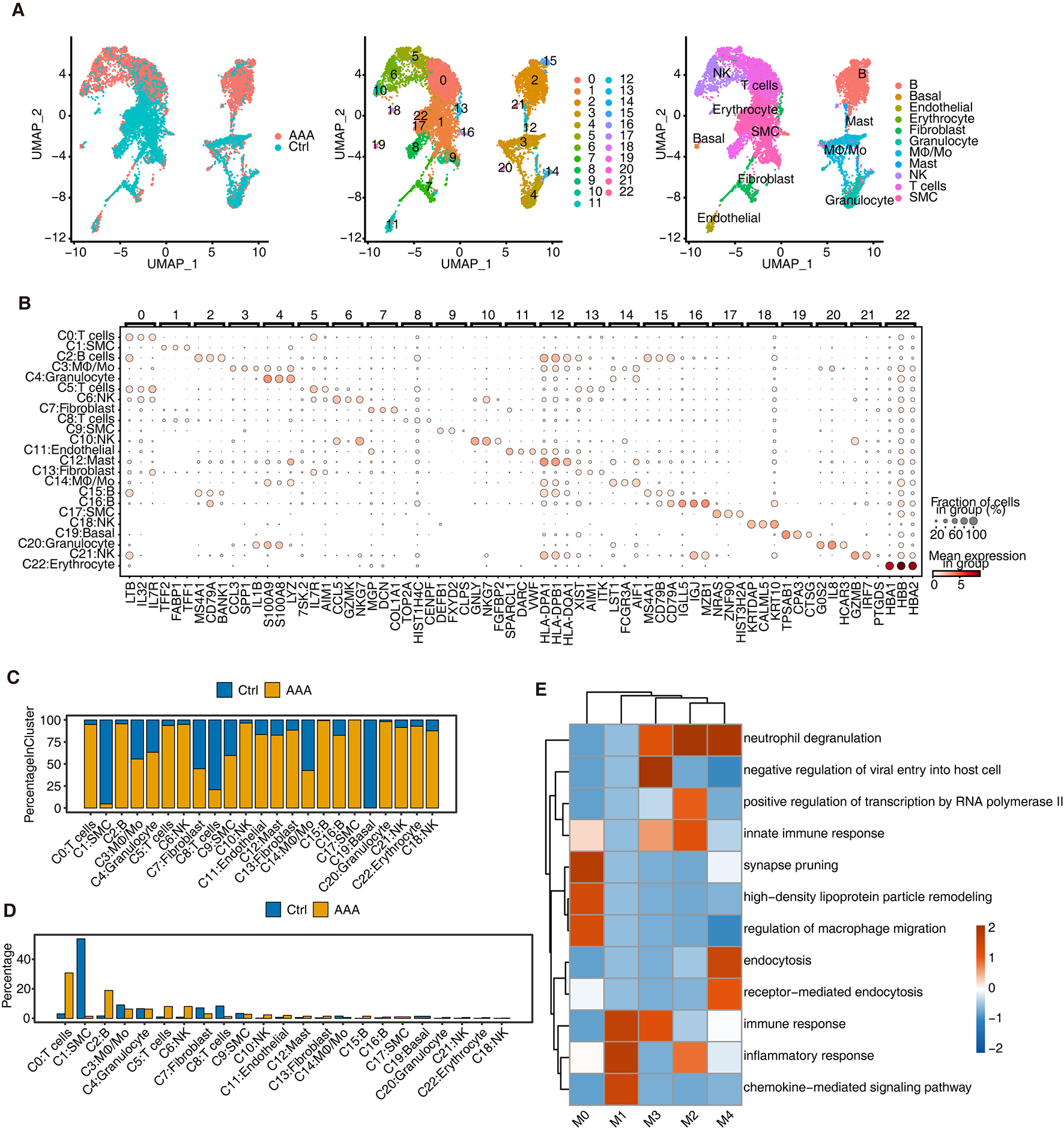

Supplement: Supplementary Figure 4 — Single-cell RNA sequencing (ScRNA-seq) of human abdominal aortic aneurysm (AAA) showed the increase of macrophage cells but not monocytes. (A) Uniform manifold approximation and projection (UMAP) plot of composite single-cell transcriptomic profiles from all human AAA and control groups. Colors indicated cell clusters along with annotations. (B) Dot plot showing expression of top 3 marker genes in each cell type. (C) Stacked bar plot showing the relative proportions of cell clusters in human AAA and control group. (D) Bar plot comparing the proportions of cell populations of cell clusters within each sample group. (E) Gene ontology enrichment analysis of biological processes of marker genes of each subgroup of macrophage/monocyte cells. Top 3 terms were selected for each cluster and heatmap shows the enrichment q-value of these terms (scaled by column). [file Image_4.TIF]

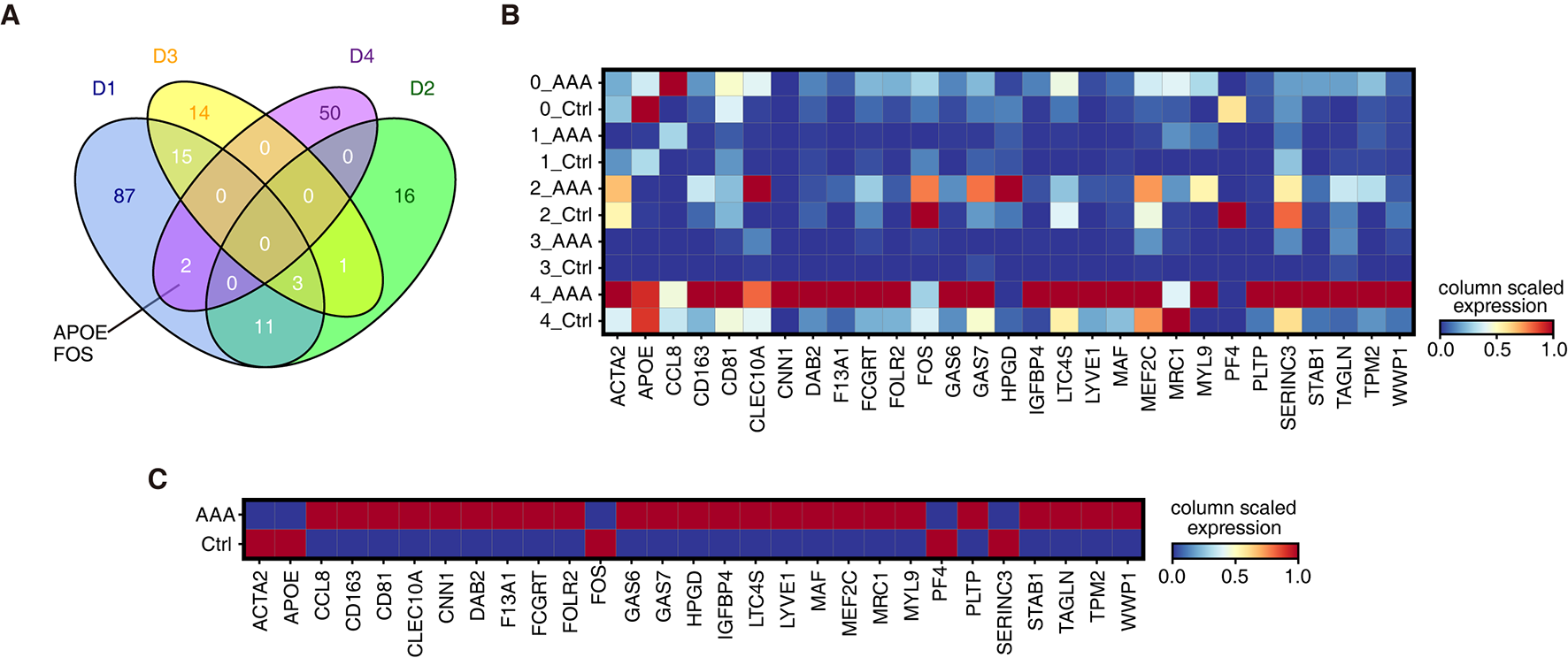

Supplement: Supplementary Figure 5 — IL-1B and THBS1 were most conserved regulated genes in macrophages/monocytes (Mφ/Mo) during abdominal aortic aneurysm (AAA) development. (A) Venn diagram showing the co-downregulated genes comparing AAA Mφ/Mo with control group from four datasets (D1–D3: mouse; D4: human). (B) Unsupervised clustering heatmap showing relative expression (column scaled) levels of downregulated genes at least in two datasets split by human AAA and control group. (C) Unsupervised clustering heatmap showing relative expression (column scaled) levels of downregulated genes at least in two datasets showed in panel (A), cells were split by different subgroups of human AAA and control group. [file Image_5.TIF]
